# Combining DNA methylation and RNA sequencing data of cancer for supervised knowledge extraction

**DOI:** 10.1186/s13040-018-0184-6

**Published:** 2018-10-25

**Authors:** Eleonora Cappelli, Giovanni Felici, Emanuel Weitschek

**Affiliations:** 1 Department of Engineering, Roma Tre University, Via della Vasca Navale, 70, Rome, 00146 Italy; 2 Institute of Systems Analysis and Computer Science, National Research Council, Via dei Taurini, 19, Rome, 00185 Italy; 3 Department of Engineering, Uninettuno University, Corso Vittorio Emanuele II, 39, Rome, 00186 Italy

**Keywords:** Classification, Next generation sequencing, RNA sequencing, DNA methylation, Cancer

## Abstract

**Background:**

In the Next Generation Sequencing (NGS) era a large amount of biological data is being sequenced, analyzed, and stored in many public databases, whose interoperability is often required to allow an enhanced accessibility. The combination of heterogeneous NGS genomic data is an open challenge: the analysis of data from different experiments is a fundamental practice for the study of diseases. In this work, we propose to combine DNA methylation and RNA sequencing NGS experiments at gene level for supervised knowledge extraction in cancer.

**Methods:**

We retrieve DNA methylation and RNA sequencing datasets from The Cancer Genome Atlas (TCGA), focusing on the Breast Invasive Carcinoma (BRCA), the Thyroid Carcinoma (THCA), and the Kidney Renal Papillary Cell Carcinoma (KIRP). We combine the RNA sequencing gene expression values with the gene methylation quantity, as a new measure that we define for representing the methylation quantity associated to a gene. Additionally, we propose to analyze the combined data through tree- and rule-based classification algorithms (C4.5, Random Forest, RIPPER, and CAMUR).

**Results:**

We extract more than 15,000 classification models (composed of gene sets), which allow to distinguish the tumoral samples from the normal ones with an average accuracy of 95%. From the integrated experiments we obtain about 5000 classification models that consider both the gene measures related to the RNA sequencing and the DNA methylation experiments.

**Conclusions:**

We compare the sets of genes obtained from the classifications on RNA sequencing and DNA methylation data with the genes obtained from the integration of the two experiments. The comparison results in several genes that are in common among the single experiments and the integrated ones (733 for BRCA, 35 for KIRP, and 861 for THCA) and 509 genes that are in common among the different experiments. Finally, we investigate the possible relationships among the different analyzed tumors by extracting a core set of 13 genes that appear in all tumors. A preliminary functional analysis confirms the relation of part of those genes (5 out of 13 and 279 out of 509) with cancer, suggesting to focus further studies on the new individuated ones.

**Electronic supplementary material:**

The online version of this article (10.1186/s13040-018-0184-6) contains supplementary material, which is available to authorized users.

## Introduction

Next Generation Sequencing (NGS) techniques have revolutionized the sequencing of genomes, producing large quantities of DNA and RNA data [1–4]. This abundance of data allows us to perform analyses on the genetic makeup of human subjects, studying the predisposition to diseases like cancer [5–8]. NGS techniques are not only applied to DNA sequencing [9], but also to other types of experiments, e.g.: transcriptome profiling (RNA sequencing) [10, 11], microRNA sequencing (miRNA-seq) [12], protein-DNA interactions (Chip-Seq) [13], identification of Copy Number Variation (CNV) [14], and characterization of the epigenome or chemical changes in the DNA (DNA methylation) [15–17].

In this work, we are going to focus on DNA methylation and RNA sequencing, as these two NGS experiments have been proven to play an important role in knowledge discovery in cancer [18–25].

DNA methylation is one of the most studied epigenetic changes in human cells. The changes in DNA methylation patterns are crucial in the development of diseases and in many forms of cancer [26–31]. Most NGS methods are based on bisulfite conversion to determine the percentage of methylated cytosines in a CpG island. This measure is called *beta value* [32], and is defined as the ratio between the methylated allele intensity and the overall intensity. For more details about the DNA methylation experimental techniques the reader may refer to [17, 33].

RNA sequencing is a next generation sequencing technique for the analysis of the transcriptome and its quantification. Four main methods for measuring gene expression are used in practice: i) Reads Per Kilobase per Million mapped reads (RPKM)[10]; ii) Fragments Per Kilobase per Million mapped (FPKM) [34]; iii) RNA-Seq by Expectation-Maximization (RSEM) [11, 35]; iv) Transcripts Per Kilobase Million (TPM) [36]. For further details about RNA sequencing, we point the reader to [37], where the authors perform a comprehensive overview of this NGS technique.

In this work, we define *NGS data* the information extracted from a NGS experiment (i.e., Chip-sequencing, DNA methylation, DNA sequencing, RNA sequencing), e.g., the counts of the reads that map on given list of genes in RNA sequencing. We define *NGS meta data* the information related to the NGS experiment and the sequenced tissue, e.g., the tissues status (tumoral, normal), or the sequencing depth. We define *NGS data integration* the procedure of joining different experiments (possibly extracted from heterogeneous databases) sharing common features (e.g., same disease / patient under study) in order to extract knowledge. The aim of integration is to aggregate genomic data in an unique schema that provides querying capabilities for retrieving data from a multitude of heterogeneous experiments and databases. Heterogeneous data are the first problem of NGS, because the structure of data is different in diverse experiments and can be different in diverse databases. Therefore, the term integration in NGS data can have different meanings [38]. On one hand, we consider integration for a need to have a uniform language that facilitates the access to different genomic databases. On the other hand data heterogeneity is caused by the experiment types and by the information that they bring. It is worth noting that dis-uniformity of the data schema is present not only when considering different databases, but also when dealing with a single one. We distinguish four conditions, where NGS data integration can be performed: (i) different databases represent the same NGS experiment (e.g. RNA-Seq) with different data schemas; (ii) different experiments (e.g., DNA methylation and RNA-Seq) in distinct databases; in this case there are two different data schemas, because the experiments need a different representation, but no standardization of the schemas is defined that allows the access to these experiments; (iii) the same problem exists even in the same databases, which contains different experiments and different data representation schemas. Finally, we consider an ideal case (iv) where a previously defined schema standardization allows to integrate different experiments that come from different databases or from the same database, and it allows also to provide interoperability between the same experiments but with different schemas. An example of this type of standardization is provided by [39] with the Genomic Data Model (GDM) that supports many NGS formats.

In order to have access to the right resources, it is necessary to define standard schemas of these data to avoid redundant information overlaps. Several efforts have been made on NGS data formats and standards. The authors of [40] provide the reader with an overview of the most widespread data formats for NGS and describe a set of standardization approaches for them. In [41] the NCBI Entrez search and retrieval system used at the National Center for Biotechnology Information to access distributed heterogeneous data is described. Also the authors of [42] present a text search engine to access data resources in the European Bioinformatics Institute (EMBL-EBI) and to help understand the relationship between different data types. Other implementations for bioinformatics data integration include retrieval systems like SRS [43] and integration tools for information fusion such as BioData Server [44]. The integration of genomic data involves multiple fields, i.e., bioinformatics, statistics, data mining, and classification. But the question is, does the integration of different types of NGS experiments offer additional knowledge about a disease like cancer [45]?

In this work, we address the issue of combining RNA sequencing and DNA methylation experiments, which have different data schemas containing heterogeneous information. Our aim is to obtain a gene oriented organization of both experiments, and therefore we define a new measure on DNA methylation data called *gene methylation quantity*. We combine RNA sequencing and DNA methylation data of The Cancer Genome Atlas (TCGA) [46] and test our method on genomic data related to three types of cancer: Breast Cancer, Kidney Renal Carcinoma, and Thyroid Carcinoma.

Additionally, we analyze the combined data by means of supervised classification algorithms, extracting classification models, which are able to distinguish the samples in two classes (tumoral and normal) and which are composed of features that represent the genes related to the disease and the different NGS experiment.

In cancer research many computational methods deal with classification problems, e.g., disease characterization, prognosis, treatment response of patients, mutation pathogenicity, biomarker prediction, and sample malignancy. A recent effort has achieved good performance in the assignment of disease subtypes and malignancy labels to melanoma images with convolutional neural networks [47]. Further studies used typical machine learning methods [48], including Adaboost [49] and decision trees [50].

Among them, we focus on a new supervised learning method that is able to extract more knowledge in terms of classification models than state of the art ones, called Classifier with Alternative and MUltiple Rule-based models (CAMUR) [51]. CAMUR is designed to find alternative and equivalent solutions for a classification problem building multiple rule-based classification models. Standard classifiers tend to extract few rules with a small set of features for discriminating the samples, and interesting features may remain hidden from the researcher. Thanks to an iterative classification procedure based on a feature elimination technique, CAMUR finds a large number of rules related to the classes present in the dataset under study. CAMUR is based on: (i) a rule-based classifier, i.e., RIPPER (Repeated Incremental Pruning to Produce Error Reduction) [52]; (ii) an iterative feature elimination technique; (iii) a repeated classification procedure; (iv) a storage structure for the classification rules. The method calculates iteratively a rule-based classification model through the RIPPER algorithms [52], deletes iteratively the features that are present in the rules from the dataset, and performs the classification procedure again, until a stopping criterion is met, i.e., the classification performance is below a given threshold or the maximum number of iterations has been reached. CAMUR has been implemented specifically for case-control studies that aim to identify subjects by their outcome status (e.g., tumoral or normal). In these data, the features correspond to the gene expressions of the samples, the classes to the investigated diseases or conditions (e.g., tumoral, normal). The extracted knowledge by CAMUR consists in a set of rules composed of a given number of genes that might be relevant for a disease. CAMUR also includes an offline tool to analyze and to interpret the computed results. Thus the software consists of two parts: (i) The Multiple Solutions Extractor (MSE), which corresponds to the implementation of the iterative classification algorithm (i.e., for each iteration it deletes the selected features, performs the classification, and saves the extracted models); (ii) The Multiple Solutions Analyzer (MSA), a graphical tool for analyzing and interpreting the obtained results. CAMUR is available at http://dmb.iasi.cnr.it/camur.php as stand alone software; for a comprehensive description we point the reader to [51].

In this work, thanks to the application of machine learning algorithms, we show the advantage of combining DNA methylation and RNA sequencing data, i.e., the increase of extracted knowledge resulting in combinations of genes from both experimental strategies. Finally, we study the three types of cancer and identify sets of relevant genes. The intersection of them results in a smaller set of genes that should be considered for further investigation.

## Methods

In this section, we discuss the methods used to combine the genomic experiments (RNA sequencing, DNA methylation) and the classification algorithms used to extract knowledge from them. We start by describing the source where we extract the data.

### Data source: the Cancer Genome Atlas

The Cancer Genome Atlas (TCGA) [46] is a project that aims to create a major repository for cancer, including NGS experiments, to improve the ability to diagnose, treat and prevent cancer through a better understanding of the genetic basis of this disease. The TCGA database contains the genomic characterization and analysis of 33 types of cancer. Tissue samples are processed through different types of techniques such as gene expression profiling (i.e., RNA sequencing and microarrays); profiling of methylated DNA (i.e., DNA methylation obtained both with NGS techniques and microarrays); profiling of microRNA (i.e., miRNA sequencing); whole genome sequecing (i.e., DNA sequencing). We rely on the latest TCGA data release available at The Genomic Data Commons platform (http://gdc.cancer.gov/).

In TCGA each tissue of DNA methylation is represented with a list of following fields: gene symbol, chromosome and genomic coordinates (where the methylation occurs), and its beta value (methylation values). RNA sequencing data instead contains information on the RSEM values [11] measured on the considered genes. It is worth noting that our approach handles gene expression data of RNA sequencing, which has been previously normalized, DNA methylation data containing the beta value, and can be used to treat also DNA methylation and RNA sequencing data of different pathologies.

### Data processing and combination

**Fig. 1:**
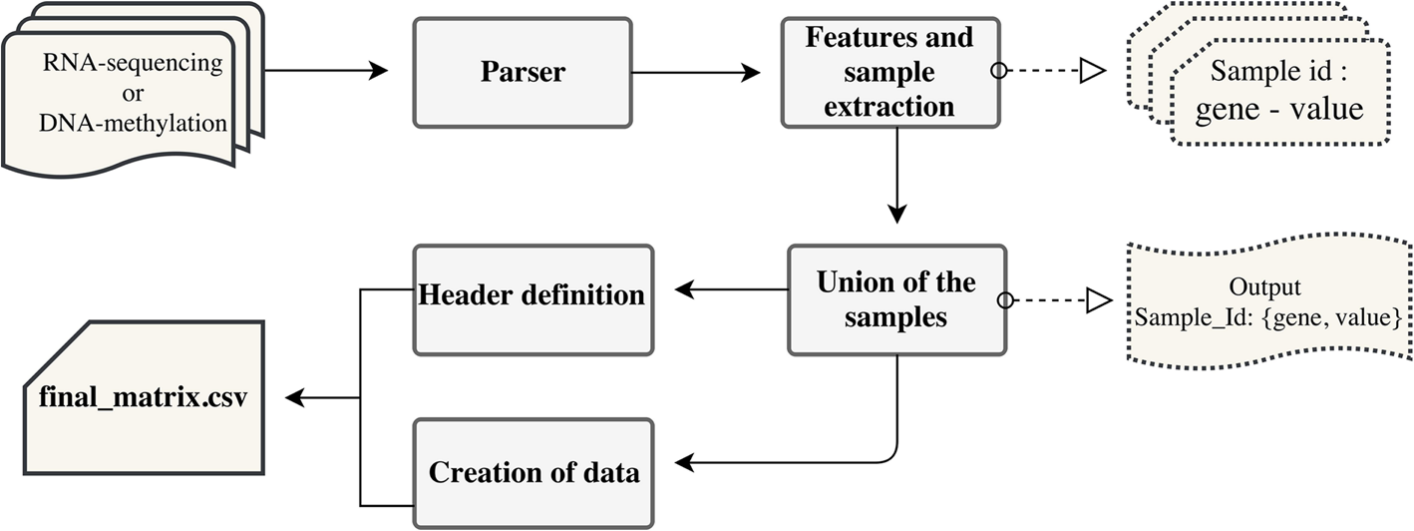
The flowchart of the computational steps for creating the RNA sequencing and DNA methylation matrices. The first step represents the parsing of the input dataset of TCGA. The samples are read for the extraction of the features (genes) and their related values, which are the gene expression measures in case of RNA sequencing, or the methylation quantities for each gene in case of DNA methylation. Subsequently, the samples and the related gene-value pairs are unified in a single file. From this file the header (columns) and the values (rows) of the matrix are created. In the final matrix (comma separated values format), the header reports all the genes, while the rows are identified by the sample id and report the related values

We create data matrices of RNA sequencing and DNA methylation experiments in the following way. Consider *n* samples (tissues) each one with *m* features (genes) and a class label (condition), which indicates whether the sample is normal or tumoral. A data matrix is composed by *n* vectors as *F*_*i*_ = (*f*_*i*,1_,*f*_*i*,2_,...,*f*_*i*,*m*_,*f*_*i*,*c*_), which represent sample *i*, where *f*_*i*,*j*_ ∈$\mathbb {R}$; *i*=1,...,*n*; *j*=1,...,*m*; *f*_*i*,*c*_ ∈ {*normal*,*tumoral*}. When considering RNA sequencing, the rows represent the samples, the columns the genes (except the last that represents the class labels) and the items of the matrix contain the RSEM gene expression values for each gene. The structure of this matrix is shown in Table 1. When considering DNA methylation, the corresponding matrix is composed by the rows that represent the samples, the columns that represent the genes, while the items contain a new measure that represent the quantity of methylation associates to each gene and that is explained in the following. Indeed, for DNA methylation TCGA encloses the beta values for each methylated site, so each sample has *s* methylated sites, *l* of them belonging to a given gene. For aggregating the methylation quantity at gene level, we consider the sum of the beta values as a measure of the overall intensity of the methylation on a gene. Let *a*_*ijh*_ be the methylation quantity associated to the sample *i* with *i*=1,..,*n*, to the gene *j* with *j*=1,....*m*, and to the methylated site *h* with *h*=1,..,*l*. Then we have $b_{i,j} = \sum _{h=1}^{l} a_{ijh}, \forall i,j$. In the following, we refer to this new measure as *gene methylation quantity*. It is worth noting that we consider the beta values of CpG sites with a related gene symbol, i.e., the symbol of the gene where the methylation occurs. If a methylation occurs on other genomic regions it is not considered in our data processing procedure, whose aim is to provide a gene oriented data organization. In Table 2 we show the structure of the DNA methylation matrix. A software tool, which performs the data extraction and the creation of the matrices, is freely available at http://bioinf.iasi.cnr.it/genint. The flowchart that reports the computational steps of the software is depicted in Fig. 1.

**Table 1:** Structure of the RNA sequencing matrix

Sample_ID	*Gene*1_*rnaSeq*_	*Gene*2_*rnaSeq*_	..	*G* *e* *n* *e* *M*_*rnaSeq*_	Class
S1	*v* *a* *l*_1,1_	*v* *a* *l*_1,2_	..	*v* *a* *l*_1, *m*_	Normal
..	..	..	..	..	..
Si	*v* *a* *l*_*i*,1_	..	..	*v* *a* *l*_*i*, *m*_	..
..	..	..	..	..	..
Sn	*v* *a* *l*_*n*,1_	..	..	*v* *a* *l*_*n*, *m*_	Tumoral

**Table 2:** Structure of the DNA methylation matrix

Sample_ID	*Gene*1_*dnaMeth*_	*Gene*2_*dnaMeth*_	..	*G* *e* *n* *e* *M*_*dnaMeth*_	Class
S1	*b*_1,1_	*b*_1,2_	..	*b*_1, *m*_	Normal
..	..	..	..	..	..
Si	*b*_*i*,1_	..	..	*b*_*i*, *m*_	..
..	..	..	..	..	..
Sn	*b*_*n*,1_	..	..	*b*_*n*, *m*_	Tumoral

**Fig. 2:**
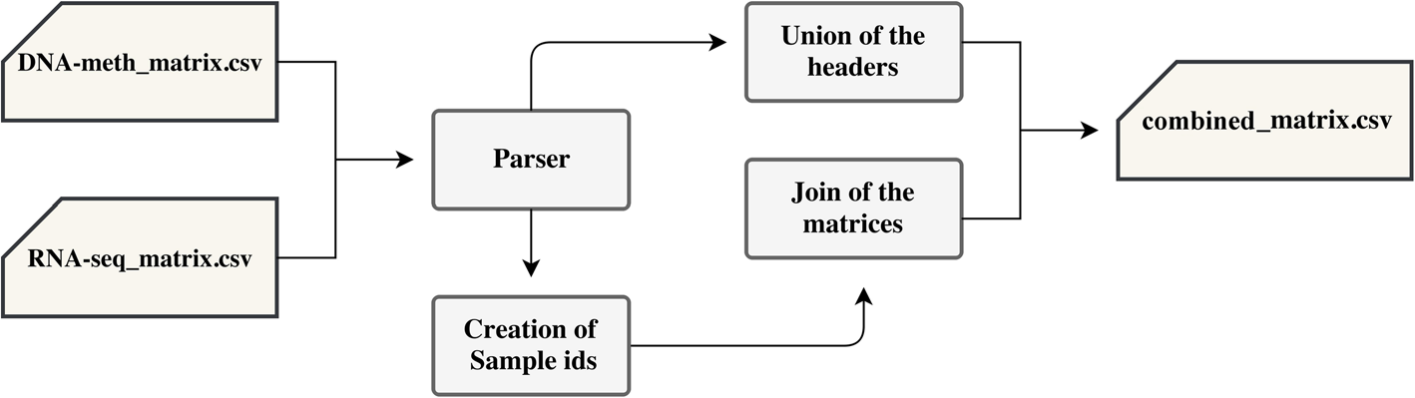
Flowchart for creating the combined matrices. Firstly, a parser reads the DNA methylation and RNA sequencing matrices in input (computed as described in Fig. 1), and sends the next elaborations to two distinct processes. A step is responsible of the creation of the full header of the combined matrix with all the genes, of both the DNA methylation and RNA sequencing. The other step takes the parsed sample IDs to modify the identification of the sample (TCGA barcode) deleting the details of the performed experiments. After the creation of the sample IDs, the join step follows: the initials matrices are joined on the modified IDs and the new rows of the matrix are created, including both gene expression and gene methylation quantity. The join defines the rows with the values of the two experiments (on which the join is made because the sample id is present in both input matrices), and also the rows with values of only one experiment (if the sample is not available in both input matrices of DNA methylation and RNA sequencing)

In order to perform our analysis on both gene oriented measures (RSEM for RNA sequencing and gene methylation quantity for DNA methylation) at the same time, we propose a combination of these two experiments by applying an intersection of the matrices on common sample IDs and a union of those not in common (this result trace over the full outer join in SQL language), keeping both experimental data and performing a union of the genes that are present in RNA sequencing and DNA methylation as features. The resulting matrix is shown in Table 3. Let *i* be the *i*-*th* sample, *j* the *j*-*th* gene, with *i*=1,..,*n* and *j*=1,..,*m* in case of a gene of the DNA methylation experiment, *j*=*m*+1,....,*z* in case of a gene of the RNA sequencing experiment. Furthermore, we have *b*_*i*,*j*_ and *val*_*i*,*j*_ ∈$\{\mathbb {R},?\}$, ∀*j*,*i*, where the “?” symbol means that there is no value associated to the gene *j* for the sample *i*. We release also a software that is able to perform the combination of the experiments, available at http://bioinf.iasi.cnr.it/genint. Finally, we report the main steps of the procedure in Fig. 2.

**Table 3:** Structure of the combined matrix

Sample_ID	*G*1_*dnaMeth*_	*G*2_*dnaMeth*_	..	*G*1_*rnaSeq*_	*G*2_*rnaSeq*_	..	Class
S1	*b*_1,1_	*b*_1,2_	..	*v* *a* *l*_1, *m*+1_	*v* *a* *l*_1, *m*+2_	..	Normal
..	..	..	..	..	..	..	..
Si	*b*_*i*,1_	..	..	*v* *a* *l*_*i*, *m*+1_	..	*v* *a* *l*_*i*, *z*_	..
..	..	..	..	..	..	..	..
Sn	*b*_*n*,1_	..	..	*v* *a* *l*_*n*, *m*+1_	..	..	Tumoral

### Analysis method

The classification that we perform has the objective of being able to determine a set of rules for each type of tumor (composed of the genes and their related values), which can define if a tissue is in tumoral or in normal condition.

It is worth noting that also in previous works [53–58] DNA methylation has been used to classify data samples (and patients) of cancer, but only a subset of single methylated sites have been used as features. Recently, the authors of [59] perform the classification task by considering all the single methylated sites in the genome with big data techniques. Conversely, we use the previously defined gene methylation quantity and not the single methylated sites. Also gene expression data of RNA sequencing has been widely used for cancer classification [51, 60–66] and proven to be effective in distinguishing normal from tumoral samples. Our aim is to combine both information in order to extract a wider knowledge and to better focus on those genes that are related to the disease.

For performing the task of knowledge discovery, we used four different classification algorithms that are briefly described in the following.

### C4.5

C4.5 [67] is an algorithm for the generation of decision trees used for classification. The algorithm takes as input a set of classified data (training set) and the output is composed by leaf nodes of the tree, which define the belonging to a class attribute. Indeed, the path from the root to a specific leaf means that all the predicates applied to the features of the sample are verified. The validity of the tree is verified on a set of labeled samples (test set), but whose class is taken into account only for verification of the class assignments. In this work, we use the J48 Java based implementation of C4.5 available in the Weka package [68].

### Random Forest

Random Forest [69] is an ensemble machine learning method that uses decision trees as basic classifiers. Each tree refers to a class and relies on a random and independent vector, which is generated with the same distribution of the others. Random Forest generates distinct decision trees, because it varies the training sets selection and the selected features for each model. The classification results of a Random Forest execution are computed by counting the votes for the most popular class predicted by the different trees and by assigning that class to the considered instance. Also for Random Forest we use its Weka [68] implementation.

### RIPPER

Repeated Incremental Pruning to Produce Error Reduction (RIPPER) [52] is an algorithm based on logic formulas, i.e., a combination of significant features with logic operators (and, or, not, <,≤, ≥, >, =) in the form of “if-then rules”. A rule-based classifier generates a set of rules for assigning a given class to each sample. In this case the features of the sample have to satisfy the conditions of the classification rule for belonging to a given class. Also for this algorithm we use the Weka [68] implementation.

### CAMUR

Classifier with Alternative and MUltiple Rule-based models (CAMUR) [51] is a multiple rule-based classifier, which extracts alternative and equivalent classification models. CAMUR combines the RIPPER algorithm with a repeated classification procedure, deleting iteratively the features that appear in the classification models from the dataset in order to extract many classification models. The iterative procedure is stopped when the classification performance is below a given threshold or a given number of iterations has been reached. In this work, we choose a particular execution mode of CAMUR (loose mode), because it allows to extract a larger number of classification models, i.e., more genes that are related to the investigated tumors. Indeed, the loose mode considers all the combinations of the features separately and without definitively removing them from the dataset. For additional details about the algorithm the reader may refer to [51].

## Results

In this section, we describe the performed experiments to test our method and the results of the classification algorithms applied to the RNA sequencing and DNA methylation data of three cancer types. We extracted all the samples of these experiments from TCGA, considering the Breast Invasive Carcinoma (BRCA), the Kidney Renal Papillary Cell Carcinoma (KIRP), and the Thyroid Carcinoma (THCA). For data extraction we used the TCGA2BED tool [70] and its TCGA data release in BED format. Every BED file is related to an experiment on a given sample identified by its TCGA barcode [46], which contains several information about the sample including the type. The sample type permits to distinguish between normal and tumoral samples, which are the two classes used for classifying the experiments. For each type of tumor, we created three data matrices, the first containing only the gene expression values (RNA sequencing), the second containing only the gene methylation quantities, and the third combining both experiments according to the procedure described in “Methods” section. Tables 4 and 5 show an example of the RNA sequencing and of the DNA methylation data matrices of BRCA. The numeric values shown in Table 2 are obtained as sums of the beta values associated with the same gene. For example for the TCGA-A7-A4SD-01A-11D-A268-05 sample, the 1.6 value, associated with the GDA gene, is the result of the sum of all the beta values of the methylation sites associated with this gene. Table 6 shows an example of the BRCA combined matrix, where each column represents a gene of the DNA methylation and then a gene of the RNA sequencing; the first row represents a sample only with data about the RNA sequencing experiment, and the second row has only data for the DNA methylation experiment, whereas the last row is an example of a sample with both experiments. Details about the combined matrices of BRCA, THCA, and KIRP tumors are summarized in Table 7, while details about the datasets are depicted in Table 8: with the column ‘Experiment’ we specify the sequencing experiment (RNA sequencing or DNA methylation), followed by the ’Cancer’ column where we indicate with a code the considered types of cancer for the two experiments. The last four columns represent the number of tumor samples, the number of normal samples, the total number of genes and the size of the matrices in MB, respectively. We performed binary classifications (two classes, normal and tumoral), and we considered three cancers with both normal and tumoral samples.

**Table 4:** Example of RNA sequencing matrix on breast cancer data

RNA sequencing	GDA_rna	SCN3A_rna	SCN3B_rna	Class
TCGA-A1-A0SD-01A-11R-A115-07	0.6	28.4	43.6	Normal
TCGA-A7-A4SD-01A-11R-A266-07	0.0	6.4	1.9	Tumoral
..	..	..	..	..
TCGA-3C-AALK-01A-11R-A41B-07	0.0	5.3786	18.2044	Tumoral

The columns represent genes, and the last shows the class. For each row, we consider the full TCGA identifier of the sample. The full identifiers is called TCGA aliquot and reports the type of the performed NGS experiment (RNA sequencing). The gene expression values are reported for all samples

**Table 5:** Example of DNA methylation matrix on breast cancer data

DNA methylation	GDA_dMeth	SCN3A_dMeth	SCN3B_dMeth	Class
TCGA-A7-A4SD-01A-11D-A268-05	1.6	2.3	2.0	Tumoral
TCGA-GI-A2C9-01A-11D-A21R-05	1.9	2.7	2.3	Tumoral
..	..	..	..	..
TCGA-3C-AALK-01A-11D-A41Q-05	3.8	2.1	3.8	Normal

Also in this case, rows are represented by the TCGA aliquot of samples, reporting the type of the performed NGS experiment (DNA methylation). The gene methylation quantity values are reported for all samples

**Table 6:** Example of combined matrix on the breast cancer data

Combined	GDA_dMeth	SCN3A_dMeth	...	GDA_rna	SCN3A_rna	Class
TCGA-A1-A0SD-01A	?	?	...	0.6	28.4	Normal
TCGA-GI-A2C9-01A	1.9	2.7	...	?	?	Tumoral
..	..	..	..	..	..	..
TCGA-A7-A4SD-01A	1.6	2.3	...	0.0	6.4	Tumoral

In the combined matrix, rows are identified by the TCGA Barcode (excluding the part that identifies the type of experiment carried out on a sample). In this way it is possible to recognize the sequenced sample with both NGS techniques (RNA sequencing and DNA methylation). In this case the matrix has as many rows as the total samples (union of RNA sequencing samples and DNA methylation samples), counting only one time those samples in common, on which both experiments were preformed

**Table 7:** Details of the number of samples in the combined matrices

	KIRP	THCA	BRCA
# RNA sequencing samples	28	9	346
# DNA methylation samples	22	8	23
# DNA methylation and RNA sequencing samples	295	563	872

# RNA sequencing samples represent the number of samples having only RNA sequencing data, # DNA methylation samples represent the number of samples having only DNA methylation data, # DNA methylation and RNA sequencing samples represent the number of samples having both information

**Table 8:** Overview of the datasets

Experiment	Cancer	Tumoral	Normal	Features	MB
RNA sequencing	BRCA	1104	114	20485	198,5
	THCA	513	59	20489	93
	KIRP	291	32	20489	52,6
DNA methylation	BRCA	799	98	20045	330
	THCA	515	56	20045	210,2
	KIRP	274	43	20045	116,9
Combined	BRCA	1114	127	40530	542,5
	THCA	515	65	40534	303,9
	KIRP	292	53	40534	171,4

### Performed tests

The data matrices of the different experiments and tumors have been analyzed with the above-mentioned classification algorithms (C4.5, Random Forest and RIPPER) through the use of the Weka software package [68]. For the application of these algorithms, we adopted a parameter tuning process to prevent overfitting and to optimize the classification results in term of accuracy. We used the Cross-Validated Parameter selection (CVParameterSelection) [71], that can optimize an arbitrary number of parameters according to input data and number of cross validation folds. We have chosen this meta-classifier for performing parameter selection by cross-validation for all our classifiers. For example, if we consider the RNA-sequencing matrix for KIRP tumor, and the different classifiers (RIPPER, C4.5 and Random Forest), we obtain the following results:

- J48 (C4.5), -C (confidence threshold for pruning.) 0.1, -M (minimum number of instances per leaf) 1, -U (use unpruned tree) false;
- JRip (RIPPER), -F (the number of folds for Reduced Error Pruning) 5, -N (the minimal weights of instances within a split) 1, -O (the number of runs of optimizations) 2 -S (the seed of randomization) 1;
- RandomForest, -I (number of iterations) 30, -K (number of attributes to randomly investigate) 0, -S (seed for random number generator) 1, -num-slots (number of execution slots) 1.

In addition, we performed the classifications with multiple rule-based models obtained by CAMUR. Therefore four different classification algorithms were applied on three data matrices (RNA sequencing, DNA methylation, and their combination) of each considered cancer, resulting in 36 different knowledge discovery analyses. For evaluating the classifiers we take into consideration the F-measure, which is defined as *F*-$measure = \frac {2P \cdot R}{P+R}$, where *R* stands for *Recall* and *P* is for *Precision*. Considering True Positives (TP) objects of a given class recognized in this class; False Positives (FP) objects recognized in a class but not belonging in this class; True Negatives (TN) objects not belonging and not recognized in a given class; False Negatives (FN) objects in a given class but not recognized in that, we can then define $Recall = \frac {{TP}}{TP+FN}$ and $Precision = \frac {TP}{TP+FP}$. We performed the tuning of parameters also for CAMUR adopting the Cross-Validated Parameter selection described above [71] for its internal RIPPER algorithm, and we finally set the execution mode to loose, the maximum number of iterations to 100, the minimum F-measure value to 0.8, and the maximum time to 30 days.

In Table 9 we show the average of the resulting F-measures for the performed classifications of each algorithm in 10-fold cross validation scheme. It is worth noting that all values are greater than 95%. Proper parameter tuning was performed with a large set of tests in order to prevent potential overfitting of the classification models.

**Table 9:** Average performance (F-measure) of the classification algorithms

Experiment	Cancer	C4.5	RIPPER	CAMUR	Random Forest
RNA sequencing	BRCA	98.5	98.1	98.2	97.3
	THCA	97.7	97.2	97.6	98.4
	KIRP	98.8	98.8	95.2	99.4
DNA methylation	BRCA	97.2	97.5	97.4	98.3
	THCA	96.1	96.3	95.1	97.0
	KIRP	97.8	96.5	98.0	99.0
Combined	BRCA	97.2	97.5	97.8	98.9
	THCA	96.4	95.2	97.2	97.3
	KIRP	98.0	96.8	98.4	98.2

The results obtained on the combined datasets are slightly lower due to the increase in features and missing values that make the job of the classification algorithms harder. In order to clarify this point we also applied the classification algorithms on the combined matrices, deleting the samples for which only one NGS experiments is available. In this way we reduced the missing values and the resulting classification performance (F-measure) improved with all the classifiers (i.e., on BRCA +0.2% with C4.5, +0.1% with RF, +1% with RIPPER; on KIRP +0.7% with C4.5, +0.8% with RF, +2,1% with RIPPER; on THCA +0.1% with C4.5, +0.5% with RF, +0.2% with RIPPER).

The performance of the algorithms are important in order to validate the classification, but the main purpose of the work is to extract more and different genes from diverse experiments. The improvement given by the classification of the combined data is that the resulting classification models do not only consider the genes and their associated values for a single experiment, but both from gene expression and DNA methylation data in a single model, providing multiple related genes. In Table 10 we show the number of genes obtained with the execution of RIPPER, C4.5 and Random Forest classification algorithms on all tumors for DNA methylation, RNA sequencing and their combination. It is worth noting that one extracted gene is in common among the three algorithms. The reason is that the algorithms operate differently and use diverse extraction functions of the models, so the extracted features are disjoint. It is important to distinguish between Random Forest that extracts multiple classification models, while RIPPER and C4.5 extract a single classification model. We obtain almost 5000 genes with Random Forest, 38 genes with RIPPER, and 26 with C4.5; we also report that 17 genes are in common between RIPPER and Random Forest, 9 between C4.5 and Random Forest, and 4 between RIPPER and C4.5. Further details and the complete list of extracted genes are available as Additional files 1 and 2 or at http://bioinf.iasi.cnr.it/genint.

**Table 10:** Number of genes obtained with the different classification algorithm

Algorithm	RNA-Seq and DNA methylation	Combination
RIPPER	26	12
C4.5	22	4
Random Forest	2098	2471

We show the number of genes obtained with RNA sequencing and DNA methylation data matrices, and the number of genes obtained thanks to the combination of the two experiments

We also investigated if the algorithms misclassify the same samples by comparing the predictions of each one. We found out that only some instances are misclassified by all the three algorithms. Further details are described in Additional file 1.

Finally, in order to prove the validity of the extracted models we performed random permutations of class membership for each classification problem and each combination. We tested if our procedure is able to extract meaningful classification models regardless of the class partition imposed on the training set. This would be verified only in the presence of a marked overfitting behavior. For validating our results and the extracted classification models, we applied the procedure to data with random permutations of class labels. This validation test was performed on 100 different random permutations for each classification problem. In particular, we obtain low values of F-measure and we report the resulting averages in Table 11. We obtain a low overall average classification accuracy on permutated data, whose values are halved when compared to the ones obtained on original data. This confirms the reliability of our classification models and suggesting the absence of overfitting when considering the correct classes.

**Table 11:** Average performance (F-measure) of the classification algorithms on random permutated class labels

Experiment	Cancer	C4.5	RIPPER	CAMUR	Random Forest
RNA sequencing	BRCA	51.1	51.6	50.2	50.9
	THCA	49.5	50.8	49.6	50.7
	KIRP	55.4	48.8	50.3	50.1
DNA methylation	BRCA	50.0	49.7	51.1	49.1
	THCA	51.1	50.2	53.2	47.9
	KIRP	50.2	49.6	52.0	50.8
Combined	BRCA	51.9	49.4	52.6	49.9
	THCA	52.4	50.7	50.1	51.3
	KIRP	50.1	50.4	50.3	50.2

We ran more than 2000 classification procedures with CAMUR, obtaining rules, literal and conjuncion lists, feature pairs and literals statistics for each tumor and each considered dataset. Detailed results are described in Additional file 1 and available at Additional file 2 or at http://bioinf.iasi.cnr.it/genint. In Table 12 we summarize the results obtained with CAMUR, in particular the table shows the total number of extracted rules and all the features (i.e., genes) that appear. In Table 13 we report the execution times, the number of iterations and the execution mode of CAMUR. The execution of the classifications procedures were run on a 4-Core 3 giga hertz Intel-7 processor with 24 gigabytes RAM and Linux Debian Kernel Version 2.6.h-amd64. The classifications obtained with the implementations of C4.5, Random Forest, and RIPPER algorithms, are executed with two software tools available at http://bioinf.iasi.cnr.it/genint. In Table 14 we report the execution times of the classification procedure for each tumor in 10-fold cross-validation sampling scheme [72]. Conversely to CAMUR, the execution times are in the order of minutes, because those algorithm extract just a single classification model. We also compared the execution times of Random Forest to those of CAMUR, which extract both multiple solutions. We note that CAMUR has higher running times than Random Forest, which are in the order of hours for CAMUR and in the order of minutes for Random Forest. We can justify this differences by considering the amount of logic formulas extracted from both classifiers, indeed CAMUR extracts many more rule-based models w.r.t. Random Forest tree-based ones.

**Table 12:** Rules and genes obtained with CAMUR

Experiment	Cancer	Rules	Genes
RNA sequencing	BRCA	1866	920
	THCA	1880	695
	KIRP	3	2
DNA methylation	BRCA	2658	1543
	THCA	3778	1918
	KIRP	159	53
Combined	BRCA	895	1045
	THCA	3703	1450
	KIRP	310	88

This results summarize the obtained output for each considered tumor and experiment

**Table 13:** Timing of the CAMUR executions, number of iterations and execution mode

Experiment	Cancer	CAMUR_time	Iterations	Mode
RNA sequencing	BRCA	14d:20h:59m:20s	60	Loose
	THCA	05d:04h:00m:51s	100	Loose
	KIRP	00d:00h:01m:22s	100	Loose
DNA methylation	BRCA	29d:00h:21m:19s	44	Loose
	THCA	29d:00h:19m:52s	39	Loose
	KIRP	00d:00h:25m:51s	100	Loose
Combined	BRCA	29d:20h:21m:25s	7	Loose
	THCA	07d:20h:53m:16s	100	Loose
	KIRP	00d:01h:34m:08s	100	Loose

We specified different maximum number of iterations according to the computation time, 80% as minimum threshold value for the classification reliability, and loose as execution mode. It is worth noting that only 7 iterations in 29 days have been performed for the combined matrix of BRCA, because the extracted classification models are composed of a high number of genes

**Table 14:** Execution time of C4.5, RIPPER and Random Forest algorithms

Experiment	Cancer	C4.5_time	RIPPER_time	RandomForest_time
RNA sequencing	BRCA	04m:07s	09m:09s	00m:48s
	THCA	01m:28s	02m:30s	00m:30s
	KIRP	00m:27s	00m:46s	00m:16s
DNA methylation	BRCA	02m:53s	06m:10s	00m:37s
	THCA	01m:34s	03m:12s	00m:34s
	KIRP	00m:45s	01m:02s	00m:22s
Combined	BRCA	06m:31s	10m:20s	6m:38s
	THCA	01m:58s	3m:35s	00m:28s
	KIRP	01m:10s	01m:45s	02m:55s

## Discussion

This section is organized as follows. Firstly, we provide evidence of proof about our new defined measure called gene methylation quantity. Then, we discuss the correlation among RNA sequencing and DNA methylation. Finally, we focus on the extracted knowledge, provide an overview of it, and, in order to prove the validity of the obtained classification models, we considered two external breast cancer RNA sequencing datasets of Gene Expression Omnibus (GSE56022 and GSM1308330) for identifying the diseased samples.

### Gene methylation quantity

In previous studies, efforts have been made for aggregating DNA methylation at gene level. In [73] a methylation index is defined as the mean percent methylation across all CpG sites in the gene. In [74] another methylation index is defined as the ratio of methylated and unmethylated copy numbers measured by absolute quantitative assessment of methylated alleles. Our measure differs from previous attempts to represent DNA methylation at gene level, because it takes into account both the number and the values of methylated sites for each gene. In order to validate the gene methylation quantity we provide a qualitative and a quantitative explanation: (i) the defined gene methylation quantity index represents how much a gene is methylated, because it is defined as the sum of the methylation values of the sites that are within the genomic coordinates of the gene, therefore if the gene methylation quantity is low/high, than the gene will be low/high methylated; (ii) we have shown that four classification algorithms are able to successfully distinguish tumoral from non tumoral samples by considering the gene methylation quantities as features. In addition, the index provides a gene oriented data representation of the DNA methylation experiment.

### Correlation between DNA methylation and RNA sequencing

An interesting problem is to investigate if there is correlation between gene expression and DNA methylation. The authors of [75–80]) address the question if there is correlation between the expression values and the methylated sites of a gene in cancer data and prove that a correlation exists only for a few set of genes. Specifically for the Breast Invasive Carcinoma, in [81] the correlation between DNA methylation and gene expression of almost 3000 genes is discussed, and in [82] it is shown how the CpG-SNP (partnership between DNA methylation and Single Nucleotide Polymorphism) pairs are strongly associated with differential expression of genes. Indeed, DNA methylation has been related also to mutations, and it has been proven that Single Nucleotide Polymorphism at specific loci can result in different patterns of DNA methylation [83].

### Tree-based classification models of C4.5

We extracted a classification model for each experiment and each cancer with C4.5, resulting in 9 decision trees composed of 26 genes (16 for DNA methylation and 10 for RNA sequencing). We show some examples on the Kidney Renal Papillary Cell Carcinoma (KIRP) data, in Table 15 we report the RNA sequencing decision tree, and Table 16 shows it for the combined data. The classification models on the other tumors and experiments are described in Additional file 1 and are available at Additional file 2 or http://bioinf.iasi.cnr.it/genint. In the leaves of the trees the total weight of instances reaching that leaf, and the total weight of misclassified instances are specified. In each leaf a fractional weight representing the instances with a missing value is considered. We can then see how missing values are handled by comparing the Tables 15 and 16. In Table 15, the weights of instances are integers, whereas in Table 16 weights are all fractional values, due to the fact that in the combined matrix most of the instances contain missing values. As we can see the model obtained from the combined data provides additional knowledge in the resulting classification rules, compared to that obtained from RNA sequencing data. In particular, the first rule of the model in Table 15 is enriched with additional rule conditions on the genes of the DNA methylation data, as shown in Table 16.

**Table 15:** The decision tree for full training set, obtained from the RNA sequencing KIRP data matrix, with 319 correctly classified instances and 4 incorrectly classified instances

UMOD_rnaSeq ≤2370.6675: tumoral (291.0)
UMOD_rnaSeq >2370.6675: normal (32.0)
Number of leaves: 2
Size of the tree: 3

**Table 16:** The decision tree for full training set, obtained from the combined KIRP data matrix, with 338 correctly classified instances and 7 incorrectly classified instances

UMOD_rnaSeq ≤2370.6675
∥ VMP1_dnaMeth ≤5.468451: tumoral (291.59/1.8)
∥ VMP1_dnaMeth >5.468451: normal (19.23/2.11)
UMOD_rnaSeq >2370.6675: normal (34.18/0.1)
Number of Leaves: 3
Size of the tree: 5

The two experiments are considered, then tumor and normal tissues are defined, both by RNA sequencing RSEM measures and DNA methylation beta values

Finally, we validated the tree-based RNA-sequencing classification models on two external datasets extracted from Gene Expression Omnibus (GSE56022 and GSM1308330), obtaining 90% correct classification.

### Tree-based classification models of Random Forest

We applied Random Forest to all matrices, extracting 9 classification models, each one composed of 30 trees. The total number of genes obtained is 2301 for RNA sequencing and 2574 for DNA methylation of which 306 are in common between two experiments. Thanks to their combination, we extracted 2471 genes with the execution of this algorithm. As example, we show in Table 17 a random tree obtained with the application of Random Forest on the DNA methylation matrix of Breast Invasive Carcinoma (BRCA) data.

**Table 17:** model A tree of the classification model for full training set, obtained by the execution of Random Forest on DNA methylation data of Breast Invasive Carcinoma

DNM2_dnaMeth <3.68
∥ CRYAB_dnaMeth <2.01
∥ ∥ AUNIP_dnaMeth <1.51 : tumoral (27/0)
∥ ∥ AUNIP_dnaMeth ≥1.51
∥ ∥ ∥ NPY_dnaMeth <4.85
∥ ∥ ∥ ∥ SACM1L_dnaMeth <2.65
∥ ∥ ∥ ∥ ∥ LINC00336_dnaMeth <4.05 : tumoral (2/0)
∥ ∥ ∥ ∥ ∥ LINC00336_dnaMeth ≥4.05 : normal (8/0)
∥ ∥ ∥ ∥ SACM1L_dnaMeth ≥2.65 : normal (76/0)
∥ ∥ ∥ NPY_dnaMeth ≥4.85
∥ ∥ ∥ ∥ PLEKHM2_dnaMeth <20.49 : tumoral (9/0)
∥ ∥ ∥ ∥ PLEKHM2_dnaMeth ≥20.49 : normal (5/0)
∥ CRYAB_dnaMeth ≥2.01 : tumoral (114/0)
DNM2_dnaMeth ≥3.68
∥ SPRYD4_dnaMeth <1.3 ∥ ∥ DAP3_dnaMeth <2.21
∥ ∥ ∥ MYOG_dnaMeth <11.05 : tumoral (646/0)
∥ ∥ ∥ MYOG_dnaMeth ≥11.05
∥ ∥ ∥ ∥ GMEB2_dnaMeth <9.57 : tumoral (1/0)
∥ ∥ ∥ ∥ GMEB2_dnaMeth ≥9.57 : normal (1/0)
∥ ∥ DAP3_dnaMeth ≥2.21 : normal (1/0)
∥ SPRYD4_dnaMeth ≥1.3
∥ ∥ LBX1-AS1_dnaMeth <10.63 : normal (5/0)
∥ ∥ LBX1-AS1_dnaMeth ≥10.63 : tumoral (2/0)
Size of the tree: 25

The full output is composed by 30 trees with different sizes with multiple leaves containing also the total weight of instances

The Random Forest algorithm is particularly suited for knowledge extraction on combined data (which presents a high number of features), because of its randomized and multiple model extraction.

For the validation of the classification models on the external datasets GSE56022 and GSM1308330, we consider all trees generated for the RNA-sequencing experiment, and the samples are classified with an average accuracy of 80%.

### Rule-based classification models of RIPPER

The RIPPER algorithm provides 9 rule-based classification models composed of 38 genes, 22 for DNA methylation and 16 for RNA sequencing. Below we show some examples of the rule-based classification models obtained with the RIPPER algorithm on the Kidney Renal Papillary Cell Carcinoma (KIRP). We show some rules for the DNA methylation dataset in Table 18, and for the combined dataset in Table 19. For example the rule depicted in Table 19 can be interpreted as: classify the considered sample into normal, if the gene methylation quantity of MAP3K11 is lower-equal then 12.3 and the one of PIP5KLI is greater-equal then 2.1 or the RSEM RNA-Seq value of NELL1 is greater-equal then 437.3. Conversely, assign the sample to the tumoral class. The reader may find all the classification models in http://bioinf.iasi.cnr.it/genint.

**Table 18:** Rule-based model for full training set, obtained from the DNA methylation KIRP data matrix, with 306 correctly classified instances and 11 incorrectly classified instances

(MAP3K11_dnaMeth ≤12.3) and (PIP5KL1_dnaMeth ≥2.1) → class=normal
→ class=tumoral
Number of Rules: 2

**Table 19:** Rule-based model for full training set, obtained from the combined KIRP data matrix, with 334 correctly classified instances and 11 incorrectly classified instances

(MAP3K11_dnaMeth ≤12.3) and (PIP5KL1_dnaMeth ≥2.1) → class=normal
(NELL1_rnaSeq ≥437.3) → class=normal
→ class=tumoral
Number of Rules: 3

Also in this case, features of both experiments appear in the extracted rule

The rules in the resulting model obtained from the combined matrix, confirm the added value. In this model we can find the same rule obtained from the single DNA methylation data, enriched with a new rule-based on a feature derived from RNA sequencing data.

We also applied the rule-based RNA-sequencing model extracted on the Gene Expression Omnibus datasets, obtaining a correct classification rate of 90% on average.

### Rule-based classification models of CAMUR

By running more than 2000 classification procedures, we extracted 15.252 rules composed of 1758 genes from RNA sequencing and 3655 genes from DNA methylation. From those genes 509 are in common in both experiments. The reader may find the gene lists in Additional file 1 or at http://bioinf.iasi.cnr.it/genint. In this subsection, we show some example of the rules obtained through the execution of CAMUR on the Thyroid Carcinoma (THCA) data. CAMUR extracts many multiple classification models (available at http://bioinf.iasi.cnr.it/genint), as example we report only those with the highest level of accuracy in Table 20.

**Table 20:** Three classification models shown for full training set, of DNA methylation experiment for Thyroid Carcinoma, with about 100% level of accuracy

(TMEM127_dnaMeth ≥1.99) and (IRGM_dnaMeth ≥1.79) and (SCN3A_dnaMeth ≥2.88) OR
(TMEM2_dnaMeth ≥1.206751) and (IL2RA_dnaMeth ≤6.32) and (NENF_dnaMeth ≥1.88) OR
(AWAT2_dnaMeth ≥3.62) and (SNORA69_dnaMeth ≤1.89)
→ class=normal
(TNFRSF12A_dnaMeth ≥0.53) and (SGK2_dnaMeth ≤11.15) OR
(TMEM127_dnaMeth ≥2.07) and (OR10J1_dnaMeth ≥2.96) and (SCN3A_dnaMeth ≥2.92)
→ class=normal
(TNFRSF12A_dnaMeth ≥0.53) and (CDKN1C_dnaMeth ≤12.92) OR
(TMEM127_dnaMeth ≥2.04) and (ADH4_dnaMeth ≤0.79) and (GFER_dnaMeth ≥2.07)
→ class=normal

The rules for the combined data classification model confirm what is derived from the model for DNA methylation data, and also provide a further classification rule-based on RNA sequencing genes that is not obtained by performing classification on data of single experiment of RNA sequencing. It is worth noting that CAMUR can be successfully adopted for knowledge extraction on combined data (which presents a high number of features), because it is able to extract multiple rule-based models.

For the validation of CAMUR we selected ten BRCA rules extracted by CAMUR and used them on the Gene Expression Omnibus datasets, noting that with these rules 80% of the samples are correctly classified, confirming the validity of the extracted models.

### Genes extracted by CAMUR

In this subsection we analyze the large quantity of classification models extracted by CAMUR, focusing on the sets of genes that occur in the rules. We summarize the common genes that appear in the different tumors and experiments with six Venn diagrams, which report the intersections of the genes among the considered tumors and the intersections among each considered experiment.

**Fig. 3:**
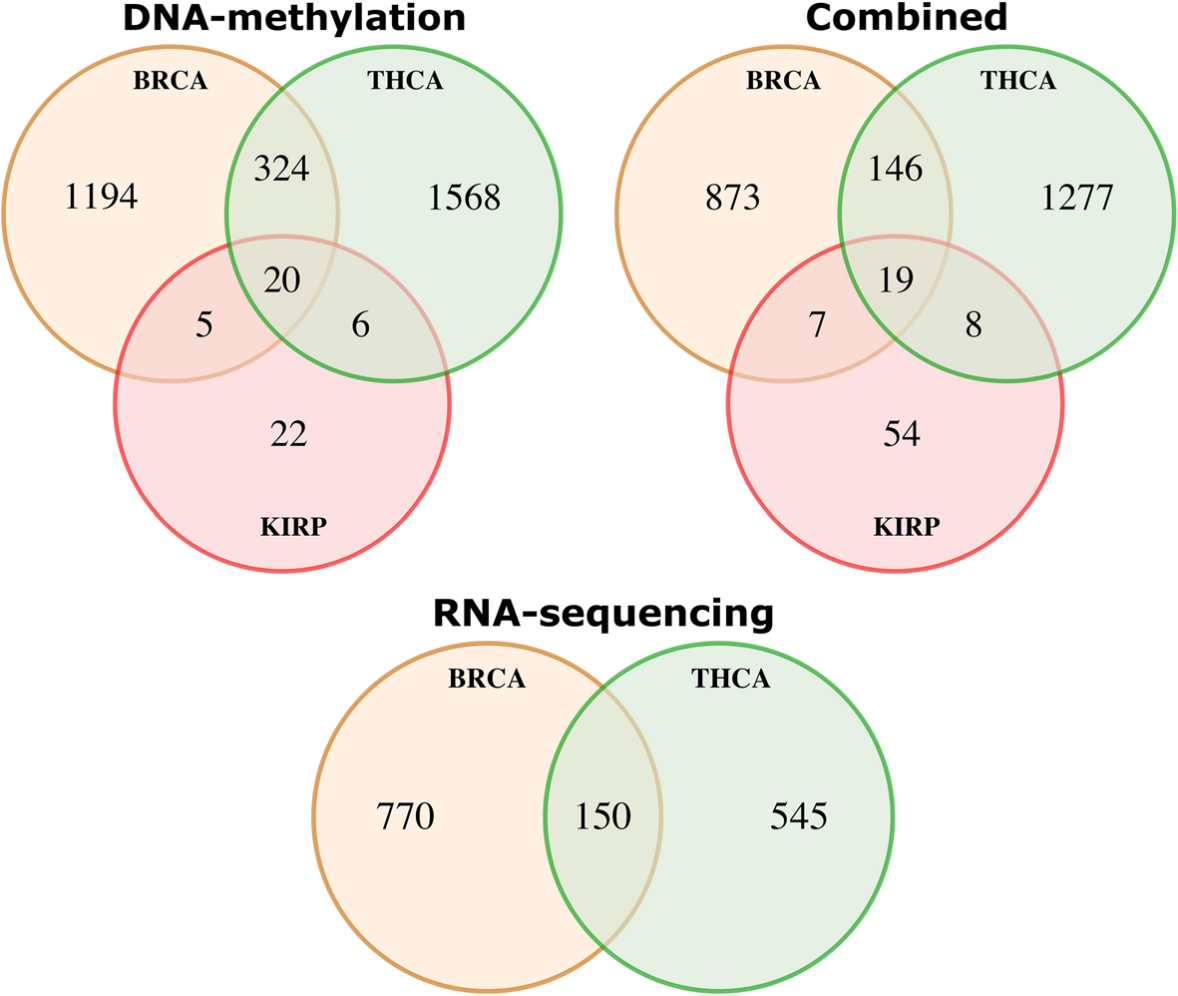
Venn diagrams representing the number of genes and their intersection that appear in the DNA methylation experiments, RNA sequencing experiments and in the combined experiments. In the DNA methylation Venn diagram, THCA, BRCA and KIRP sets have 20 common genes, 6 genes are in common between THCA and KIRP, 5 genes between BRCA and KIRP, and 324 between THCA and BRCA. In the RNA sequencing Venn diagram, BRCA and THCA have 150 genes in common, whereas the intersections with the KIRP set of genes are empty, therefore they are not represented.In the combined Venn diagram THCA, BRCA and KIRP sets have 19 common genes, 8 genes are in common between THCA and KIRP, 7 genes between BRCA and KIRP, and 146 between THCA and BRCA

In Fig. 3 we show the intersections between the tumors sets of genes for the DNA methylation experiment, the RNA sequencing experiment and their combination. The classification models obtained for the DNA methylation experiment of Breast Cancer (BRCA) and of Thyroid Carcinoma (THCA) result in 324 common genes, while the intersections with the sets of genes for the Kidney Renal Carcinoma (KIRP) result in fewer genes (5 for BRCA and KIRP intersection and 6 for THCA and KIRP intersection). Furthermore, 20 genes are in common between all tumors. For RNA sequencing we have 150 genes in common for the BRCA and the THCA tumors. Only 2 genes are extracted for the KIRP tumor that are not in common with the other tumors. In the combined Venn diagram 19 genes are in common between all the tumors, 146 between BRCA and THCA, and less than 10 genes for the intersection of THCA and BRCA with the KIRP set.

**Fig. 4:**
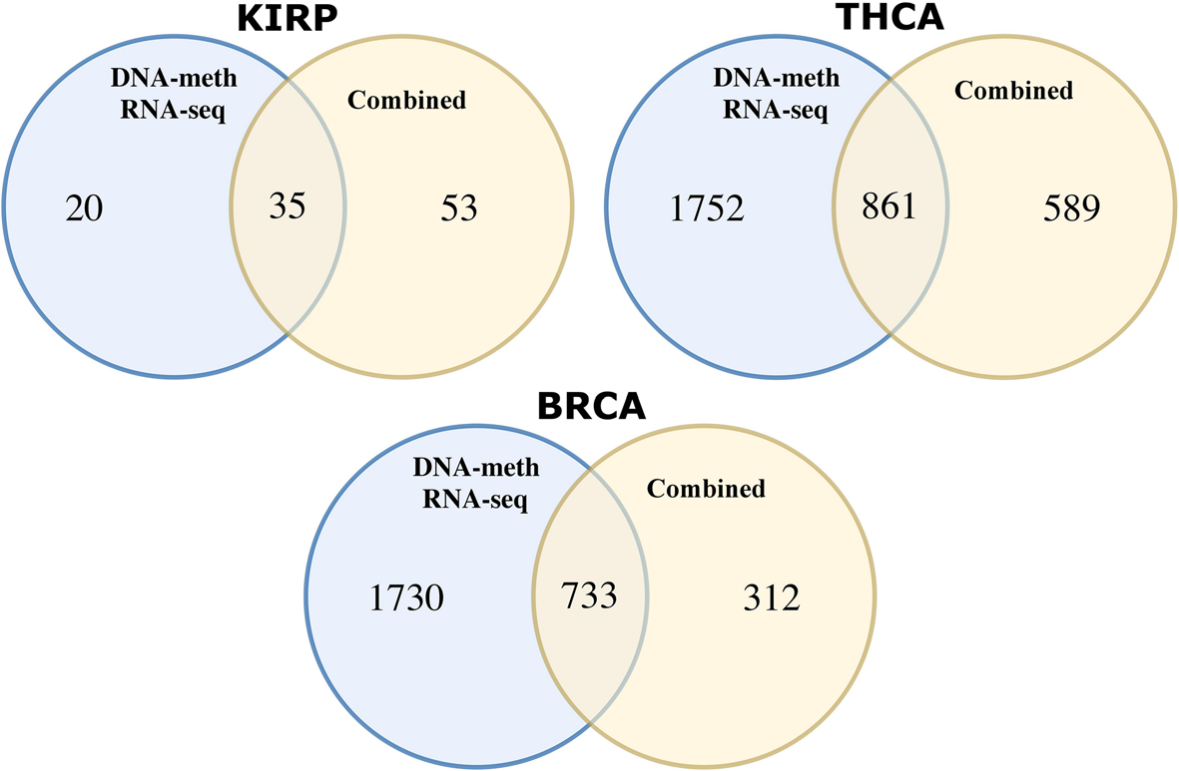
The Venn diagrams representing the number of genes and their intersection in each tumor. In KIRP 35 genes are in common between the set of genes extracted from the combined matrix and the union of the genes sets extracted from the DNA methylation and RNA sequencing matrices. We obtain 861 common genes for THCA and 733 for BRCA matrices

In Fig. 4 we consider a Venn diagram for each tumor, where the set on the left reports the number of genes calculated from the union of genes of RNA sequencing and DNA methylation experiments, and the set on the right stands for the number of genes extracted from the combined matrix. In each tumor different and common genes are extracted from the combined matrices. For example, in BRCA 733 genes are in common, and 312 are the new genes obtained thanks to the combination of RNA sequencing and DNA methylation. We report also 1730 genes that do not appear in the combined dataset of genes. For the THCA tumor 861 gene are in common, 589 belong to the combined dataset of genes, and 1752 genes are extracted from DNA methylation and RNA sequencing matrices. Finally, the KIRP diagram reports 53 new genes for the combined matrix, 35 in common, and 20 present in the union of the two experiments. In this case, the combination produced more genes than those extracted from the single experiments. The combination leads to the extraction of new genes, which are not computed when analyzing single experiments. The goal of this work is also to study the different types of cancer and identify many genes related to the disease. By performing the intersection it is possible to reduce the number of them and to focus on a number of potential oncogenes. In Additional file 2 and at http://bioinf.iasi.cnr.it/genint we provide all the gene lists that are in common and not in common for each tumor and experiment. Thanks to the intersections, we are able to detect 509 genes in common among DNA methylation and RNA sequencing experiments. From these genes we extract a subset of 13 genes, which are in common among the different tumors. In order to check biological relevance of the obtained subset of genes, we compared our result with the Entrez Gene database of NCBI [84], which provides information about oncogenes, tumor suppressor genes, the over-expression or lower-expression of gene regulation of cancer cells growth, and also hyper-methylation and hypo-methylation closely associated with the progression of cancer. This analysis led to the detection of 5 and 279 cancer related genes, from the subsets of 13 and 509 genes discussed above, respectively. Those genes are available as Additional file 2.

## Conclusion

In this work, we described the integration of Next Generation Sequencing experiments by distinguishing four cases: (i) integration of data that represent the same NGS experiments, stored in different databases with different schemas; (ii) integration of data that represent different NGS experiments, stored in different databases and with no standardization of schema allowing the access to them; (iii) integration of data that represent different NGS experiments, stored in the same database and with distinct schemas. We also individuated an ideal case for integration (iv), i.e., when a standardization of the schema is provided in order to permit the interoperability between different experiments, with different schemas in different or same databases. We proposed a method for the combination of two distinct NGS experiments with different data schemas. The NGS experiments considered in this study were DNA methylation and RNA sequencing and were extracted from TCGA. We focused on three forms of tumors, i.e. BRCA, KIRP, and THCA. We defined the data matrices, one for each NGS experiment, with samples in the rows and genes in the columns, and a third matrix for representing the combination of DNA methylation and RNA sequencing samples. In particular the objective of the combination was the creation of data matrices indexed on the genes that are related to both NGS experiments. In the RNA sequencing matrices the items are the RNA-Seq by Expectation-Maximization (RSEM) values that quantify the gene expression, whereas in DNA methylation matrices we defined a new measure based on the beta value, that we call *gene methylation quantity* for denoting the quantity of methylation associates to each gene. After the combination of RNA sequencing and DNA methylation, we proposed the application of supervised analyses. We were able to extract many classification models containing the genes and their quantification values by applying different supervised algorithms (decision trees, rule-based classifiers, and multiple rule-based ones). The classifications were performed on all matrices for each tumor, with the objective to obtain models to separate the normal from the tumoral samples. All the classification models have an accuracy greater than 95%. In particular we obtained 9 decision trees with C4.5, 9 rule-based classification models with RIPPER, and 9 classification models (each one composed of 30 decision trees) with Random Forest. Moreover, thanks to the execution of more than 2000 classification procedures with CAMUR, we extracted 15.252 classification models, from which we derived 5413 genes related to DNA methylation and RNA sequencing. 509 genes are in common among the different experiments and 13 genes among the different tumors. Through the NCBI Entrez gene database we performed functional analysis of those genes. We found 279 out of 509 and 5 out of 13 of them already marked as oncogenes. CAMUR was applied in order to extract possible oncogenes and to find new ones. Many of the extracted genes have been already classified as oncogenes, and this confirms that our method is able to identify relevant genes and justifies further analyses [85]. Indeed, we suggest as future direction a further biological investigation of the classifications models and the extracted sets of genes to confirm their relation with the considered tumors. As other future work, we suggest the application of our method to other forms of tumors. Finally, we plan to define new gene wide measures on different NGS experiments (e.g., mutations, copy number variations, chip-sequencing) in order to consider the combination of them for a comprehensive knowledge extraction.

## Additional files

Additional File 1 Description of the supplementary material. (PDF 99 kb)

Additional File 2 Detailed results. (RAR 6710 kb)
