# Supplementary material for: Combining DNA methylation and RNA sequencing data of cancer for supervised knowledge extraction
Source: BioData Min. 2018 Oct 25;11:22. doi: 10.1186/s13040-018-0184-6 (PMC6203208; doi:10.1186/s13040-018-0184-6)
Supplement: Supplementary file 1 — Description of the supplementary material. (PDF 99 kb) [file 13040_2018_184_MOESM1_ESM.pdf]

Combining DNA methylation and RNA sequencing data of cancer for supervised knowledge extraction

Supplementary Online Materials

Eleonora Cappelli, Giovanni Felici, and Emanuel Weitschek

The *supplementary data* include additional details about the experimental results. In particular, the reader may download three compressed archives and a file:

- *classification_models.zip*;
- *genes_analysis.zip*;
- *genes_analysis_weka.txt*;
- *predictions.zip*.

The related software packages and the supplementary data are available at: http://bioinf.iasi.cnr.it/genint

Classification models

The *classification_models* folder contains the classification models obtained with the execution of the algorithms C4.5 (J48), RIPPER (Jrip) and Random Forest (RF) on the considered data matrices, and outputs of CAMUR. In particular the "classification_models" folder includes:

1. the *C4.5* subfolder that contains 3 subfolders, one for each tumor, each one containing 3 text files that report the classification models obtained with the C4.5 algorithm and 3 text files that report the predictions for each obtained model.
   The files of the classification models are named experiment_Matrix_tumor_output_J48.txt, where for "experiment" we can have "DNAmeth", "RNAseq" or "INT", and for "tumor" we can have "brca", "thca", or "kirp". The files of the predictions are named experiment_Matrix_tumor_output_predictions_J48.txt, where for "experiment" we can have "DNAmeth", "RNAseq" or "INT", and for "tumor" we can have "brca", "thca", or "kirp".

2. the *RIPPER* subfolder contains 3 subfolders, one for each tumor, each one containing 3 text files that report the classification models obtained with the RIPPER algorithm and 3 text files that report the predictions for each obtained model. The files of the classification models are named
experiment_Matrix_tumor_output_Jrip.txt, where for "experiment" we can have "DNAmeth", "RNAseq" or "INT", and for "tumor" we can have "brca", "thca", or "kirp". The files of the predictions are named experiment_Matrix_tumor_output_predictions_Jrip.txt, where for "experiment" we can have "DNAmeth", "RNAseq" or "INT", and for "tumor" we can have "brca", "thca", or "kirp".

3. the *RandomForest* subfolder contains 3 subfolders, one for each tumor, each one containing 3 text files that report the classification models obtained with the Random Forest algorithm and 3 text files that report the predictions for each obtained model. The files of the classification models are named
experiment_Matrix_tumor_output_RF.txt, where for "experiment" we can have "DNAmeth", "RNAseq" or "INT", and for "tumor" we can have "brca", "thca", or "kirp". The files of the predictions are named experiment_Matrix_tumor_output_predictions_RF.txt, where for "experiment" we can have "DNAmeth", "RNAseq" or "INT", and for "tumor" we can have "brca", "thca", or "kirp".

4. the *CAMUR* subfolder contains 3 subfolders, one for each tumor, each one containing text files, which report the outputs of the CAMUR tool (i.e., the classification models, the list of the genes, the genes that appear together, etc.). The files are named
outputName_experiment_Matrix_tumor.txt, where for "outputName" we have the name of the output of CAMUR, for "experiment" and can have "DNAMeth", "RNA" or "INT", and for "tumor" we can have "brca", "thca", or "kirp".

For further details about the outputs of CAMUR the reader may refer to the user guide available at http://dmb.iasi.cnr.it/camur.php. Finally, in the "CAMUR" subfolder there is a text file that reports the execution times of CAMUR on the analyzed experiments on a 4-Core 3 giga hertz Intel-7 processor with 24 gigabytes RAM and Linux Debian Kernel Version 2.6.26-2-amd64.

Genes analysis

The *genes_analysis* folder contains the lists of genes extracted by CAMUR that are in common and not in common among the analyzed cancers (BRCA, KIRP, and THCA), and the lists of genes that are in common and not in common among the different experiments (DNA-methylation, RNA-sequencing, and the integration of them). In particular the "genes_analysis" folder includes three subfolders "tumors_genes", "experiments_genes", and "oncogenes":

1. the *tumors_genes* subfolder that contains 3 text files for each tumor (common_genes_brca.txt, common_genes_kirp.txt, common_genes_thca.txt), which report the lists of common genes between the union of the genes extracted by RNA-sequencing and DNA-methylation experiments, and the genes extracted by the integration of the two esperiments.The folder contains also 3 text files (noCommon_genes_brca.txt, noCommon_genes_kirp.txt, noCommon_gens_thca.txt), which report for each tumor the lists of not common genes between the union of the genes extracted by RNA-sequencing and DNA-methylation experiments, and the genes extracted by the integration of the two esperiments (the genes that belong to the integration are identified by the word "int"). Furthermore the folder contains a file (common_genes_between_tumors.txt), which contains a list of genes shared among the three cancers.

2. the *experiments_genes* subfolder contains 3 text files (genes_RNA-sequencing.txt, genes_DNA-methylation.txt, genes_INTegrated.txt) that report the lists of extracted genes for each experiment. Each gene is identified by the name of the tumor or tumors to which it relates (example: ST8SIA6_dnaMeth_brcathca, indicates that the gene ST8SIA6 belongs to the DNA-methylation experiment and is in common between the tumor datasets BRCA and THCA). Additionally, the folder contains a file (RNA-seq_DNA-meth_commonGenes.txt) with a list of common genes between the two experiments.

3. the *oncogenes* subfolder contains 2 text files oncogenes_in_common_between_all_tumors.txt and oncogenes_in_common_between_RnaSeq_and_DnaMeth.txt. The latter contains all the genes classified as oncogenes from NCBI that are in common between RNA sequencing and DNA methylation experiments

of all tumors. The first one contains the subset of those oncogenes that appear in the CAMUR rules of all tumors.

In addition we provide a text file, *genes_analysis_weka*, which contains the lists of genes extracted with the execution of the three classification algorithms, C4.5, RIPPER and Random Forest.

The file reports the list of file names containing the classification models that are analyzed, followed by a list of genes corresponding to RNA-sequencing experiment and a list of genes corresponding to DNA-methylation experiment. Additionally, we provide a list of genes in common, a list of genes not in common between the two experiments and a list of genes obtained thanks to the integration of RNA-sequencing and DNA-methylation experiments. For each experiment we analyzed the number of genes extracted with the execution of C4.5, RIPPER, and Random Forest on the data of the three cancers (BRCA, KIRP, and THCA). Finally, we report the gene extracted by all the three algorithms.

Predictions

The *predictions* folder contains 9 text files (DNAmeth_brca_predictions.txt, DNAmeth_thca_predictions.txt, DNAmeth_kirp_predictions.txt, RNAseq_brca_predictions.txt, RNAseq_thca_predictions.txt, RNAseq_kirp_predictions.txt, INT_brca_predictions.txt, INT_thca_predictions.txt, INT_kirp_predictions.txt), where we provide the details about the misclassified instances with the execution of the C4.5, RIPPER and Random Forest algorithms, on each data matrix.
In Table 1 we summarize the number of incorrectly classified instances and the ones that are misclassified by all three algorithms.

	RNA-sequencing			DNA-methylation			Integration		
	BRCA	THCA	KIRP	BRCA	THCA	KIRP	BRCA	THCA	KIRP
**Random Forest**	18	9	2	15	16	3	13	15	6
**RIPPER**	23	16	4	23	22	11	31	28	11
**C4.5**	18	13	4	25	22	7	35	21	7
**common**	3	2	1	9	6	2	9	3	1

Table 1: Incorrectly Classified Instances. The Table reports, for each cancer and experiment, the number of misclassified instances by C4.5, RIPPER and Random Forest algorithms. The last row shows common instances that are misclassified by all the three algorithms.
